# Reflecting on the impact of the COVID pandemic on patient management and its subsequent influence on long-term outcomes: a case–control study in the field of esophago-gastric cancer

**DOI:** 10.1186/s12957-024-03621-0

**Published:** 2025-01-14

**Authors:** Mohamed Alasmar, Nadia Matias, Norah Ali M. Alhamed, Omneya Alwani, Brogan Rudge, Terngu David Moti, Muhammad Ossama Yassin Abdelwahab, Jennifer Stockton, Charef Raslan, Jess Cairney-Hill, Mohammad Altarawni, Bilal Alkhaffaf, on behalf Greater Manchester Oesophago-Gastric Service

**Affiliations:** 1 Department of Oesophago-Gastric & Bariatric Surgery, Salford Care Organisation, Northern Care Alliance NHS Foundation Trust, Salford Royal Hospital, Manchester, UK; 2 Division of Cancer Sciences, School of Medical Sciences, Faculty of Biology, Medicine and Health, University of Manchester, Manchester, UK; 3 Department of General Surgery, Jordan University Hospital, Amman, Jordan

## Abstract

**Background:**

The delivery of cancer services changed significantly during the COVID-19 pandemic. This study aimed to describe changes in presentations, assess the change in recommendations by the MDT during the pandemic, and describe the subsequent long-term impact of these changes on survival rates in patients with EG cancer.

**Methods:**

A retrospective cohort study was designed comparing three patient groups of those referred to EG MDT in the same 6-month period pre-pandemic (PP;2019) during the initial phase of the pandemic (P1;2020) and the year after the initial phase (P2;2021). The primary aim of this study was to describe and compare deviations from the standard of care across these three timeframes. Secondary outcomes included differences in the number of new cases with early and advanced oesophageal and gastric lesions, a comparison of survival rates among the groups, and an analysis of postoperative histopathology to identify any shifts in the tumour stage across the studied periods.

**Results:**

A consistent demographic profile across these periods was maintained, but with a significant decrease in patient referrals during P1 (35.25% reduction from PP to P1 and 9.5% reduction from PP to P2), quicker ‘time to treatment’ during P1 (130.8 days in P1 vs 162 in PP and 178.9 in P2), and notable changes in treatment modalities. Additionally, we found an increased deviation from initial curative to palliative intent in the P2 group (6.4% changed in P2 vs 2.2% in PP and 3.5% in P2) primarily driven by disease progression. A further significant observation was the emergence of more aggressive tumour characteristics, particularly in the P2 group, albeit without a statistically significant difference in two-year overall survival rates among the groups (*p*-value 0.31).

**Conclusion:**

The COVID-19 pandemic significantly impacted oesophagogastric cancer care, with a reduction in patient referral rates during the initial pandemic phase and a subsequent increase in more advanced stage disease. Our findings from a major UK EG centre highlight accelerated treatment decision-making during the initial pandemic phase was possible and that standard of care was maintained. These insights provide valuable lessons for healthcare systems in managing cancer care during global health emergencies.

## Background

Sars Cov-2 (Covid-19) was classified as a pandemic by the World Health Organisation (WHO) on 12 March 2020 [1]. The subsequent clinical pressures resulted in a worldwide reconfiguration of healthcare provision whereby non-essential services were closed or reduced to free up resources for areas of increased demand [2]. Cancer services were maintained but with significant changes to how they were delivered [3]. In addition to chronic staff shortages, a lack of intensive care beds and the overall prioritisation of Covid-19 admissions were factors likely to have a negative impact on the diagnosis and treatment of cancer patients, including those with esophagogastric (EG) cancers who required complex surgical care in a high dependency setting [4].

In addition to the logistical disruption, guidance regarding cancer treatment during the pandemic changed as the pandemic progressed [5]. Most pertinently, The UK’s National Institute for Health and Care Excellence published guidance which divided the priority of systemic anticancer treatment into six priority levels, taking into account capacity issues whilst ​​balancing the risk of suboptimal cancer treatment with the risk of immunosuppression and becoming seriously ill from Covid‑19. This guidance advised that this prioritisation should be part of multidisciplinary team (MDT) discussions for cancer patients and should be clearly documented within the MDT minutes [6]. The rapid evolution of guidance over this period resulted in a significant change to the management of cancer patients from diagnosis, MDT discussions and definitive treatment options. The true impact on decision-making by MDTs during these times of unprecedented challenges remains largely unknown, as do the longer-term implications that this may have had.

This study aimed to describe changes in presentations, assess the change in recommendations by an MDT during the pandemic, and describe the subsequent impact of these changes upon survival rates in patients with EG cancer. Understanding the degree to which management decisions deviated from the ‘standard of care’ during the pandemic is vital in comprehending its contribution to short—and longer-term outcomes in this patient group.

## Methods

### Study design

This retrospective cohort study compared three patient groups: pre-pandemic (PP; March to August 2019), initial phase of the pandemic (P1; March to August 2020), and a subsequent pandemic phase (P2: March to August 2021).

Each patient group included all patients referred to the EG center who met the inclusion criteria from 1st March to 31st August (6 months) of each respective year. These groups were deliberately chosen to parallel key stages of the COVID-19 pandemic in the UK. P1 corresponds to the 6-month period coinciding with the first wave of the pandemic in the UK. In contrast, the PP and P2 groups cover equivalent 6-month periods in the year before and the year after, respectively. This methodology was adopted to prevent overlap between patient groups, aligning with the usual duration of oesophagogastric cancer treatment for clear and comprehensive analysis.

### Setting

Data was collected from a UK tertiary referral EG centre serving a regional population of 3.2 million. The centre manages the care of approximately 1000 new EG cancer diagnoses per annum.

During the COVID-19 pandemic, the centre implemented several structural changes to adapt to evolving challenges while maintaining high standards of care. Multidisciplinary team (MDT) meetings were transitioned to remote formats to minimize in-person interactions and ensure continuity. Access to staging investigations, including endoscopic ultrasound (EUS) and PET scans, was limited, particularly during the initial phase of the pandemic (P1).

### Participants

All new patients referred to the EG cancer MDT with malignancy of the oesophagus and stomach within the designated time periods for each group who met eligibility criteria were included. Participants across all groups were followed up for a minimum of two years. The following eligibility criteria for patients were applied:

- Aged 18 years and over
- Pathology: malignant lesions in the oesophagus and stomach
- Treatments and interventions: palliative and curative treatment pathways

### Variables

#### Retrospective data collection

For all groups included patient demographics (age and sex), disease data (histological type and clinical staging), MDT treatment recommendations with justification, surgical decisions (surgical plan, provision, and outcome), changes in treatment (including changes in the aim of treatment or treatment modality, and reasons for these changes), and postoperative histopathology findings (with respect to surgical patients), including tumor stage and positive resection margins, were recorded. Patient survival at two years was also evaluated to assess the overall effect of the pandemic on survival rates.

The primary aim of this study was to describe and compare deviations from the standard of care across the three groups (PP vs P1 vs P2). Secondary outcomes included differences in the number of new cases with early and advanced oesophageal and gastric lesions referred to the MDT, a comparison of survival rates among the groups, and an analysis of postoperative histopathology to identify any shifts in tumour aggressiveness and staging across the studied periods.

### Study size

This study's sample size was specifically tailored to the nature of EG cancer care, where the trajectory of diagnosis and treatment typically spans several months. As this research was undertaken in one of the UK's largest EG centers, the 6-month timeframe was also pragmatic, ensuring a sufficient volume of cases to robustly meet the study’s objectives.

### Data sources

Data was collected from the centre’s well-established (since 2000) and comprehensive electronic patient records (EPR), and MDT minutes. The study data was collected by an experienced team of clinicians in EG cancer surgery, including consultants, senior trainees and advanced care practitioners. Management intention (palliative or curative) and the justification for decisions were captured from MDT meeting minutes. The framework on which MDT decision-making was based was taken from Wathes et al. [3] who devised a system of structured keywords to track cancer patients during the pandemic. This was combined alongside a consultative and iterative process developed within the study management team to identify common reasons for decision-making (see below). Treatment intent and likely outcome were divided to curative and palliative. Any change in patient pathway due to Covid-19 was classed as “adjusted pathway”.

Reasons for curative or palliative treatment intent and modalities were categorised as follows:

- 1- (Potentially) curable disease
- 2- Metastatic or unresectable disease
- 3- Poor fitness or multiple comorbidities precluding treatment
- 4- Patient choice

Reasons for change in the initial patient pathway were classified as.

- 1- Deterioration in patient fitness
- 2- Incomplete resection
- 3- Disease progression
- 4- Patient choice

The NHS Spine platform (which updates monthly) was used to collect survival data.

### Data analysis

Descriptive statistics were used to report participant characteristics and outcomes in this study. Statistical analysis was performed using the R statistical package [7]. The Shapiro test was used to test for normality of continuous variables, the Wilcoxon test to compare non-parametric continuous variables, Pearson's chi-squared test and Fisher's exact test for categorical variables and the log-rank test for survival analysis. A *p*-value of < 0.05 was used to denote a statistically significant result.

## Results

### Demographics and basic characteristics

Table 1 describes the comparative analysis of demographics and basic characteristics of patients with oesophagogastric cancer across three distinct periods: PP(2019), P1 (2020), and P2 (2021). There was a notable decrease in the number of referrals to the MDT during the P1 period (2020: 259, 25.37%) compared to the Pre-pandemic period (2019: 400, 39.18%), with only a partial recovery in the P2 period (2021: 362, 35.46%), indicating the profound impact of the pandemic on patient referrals.

**Table 1:** Overview of patient demographics and basic characteristics in PP (2019), P1 (2020) and P2 (2021) phases

	PP (2019)	P1 (2020)	P2 (2021)	Total
Total	400	259	362	1021
Sex (*p*-value = 0.1049)				
Female	143 (35.7%)	73 (28.2%)	127 (35.1%)	343 (33.6%)
Male	257 (64.3%)	186 (71.8%)	235 (64.9%)	678 (66.4%)
Histological types (*p*-value = 0.02612)				
High-grade dysplasia	33 (8.2%)	9 (3.5%)	15 (4.1%)	57 (5.6%)
Adenocarcinoma	229 (57.3%)	161 (62.2%)	242 (66.9%)	632 (61.9%)
Squamous cell carcinoma	83 (20.7%)	53 (20.5%)	74 (20.4%)	210 (20.6%)
Unobtainable Histology	41 (10.2%)	29 (11.2%)	25 (6.9%)	95 (9.3%)
Tumour site (*p*-value = 0.004498)				
Oesophageal	316 (79.0%)	188 (72.6%)	273 (75.4%)	777 (76.1%)
Gastric	81 (20.3%)	64 (24.7%)	89 (24.6%)	234 (22.9%)
Metastatic/Unspecified	3 (0.7%)	7 (0.7%)	0	10 (1%)
Staging (*p*-value = 0.0004998)				
Stage 0	39 (9.8%)	12 (4.6%)	25 (6.9%)	76 (7.4%)
Stage 1	18 (4.5%)	13 (5%)	22 (6.1%)	53 (5.2%)
Stage 2	30 (7.5%)	23 (8.9%)	58 (16%)	111 (10.9%)
Stage 3	87 (21.7%)	50 (19.3%)	93 (25.7%)	230 (22.5%)
Stage 4	189 (47.2%)	134 (51.7%)	138 (38.1%)	461 (45.2%)
Not completely staged	37 (9.2%)	27 (10.4%)	26 (7.2%)	90 (8.8%)

**Fig. 1:**
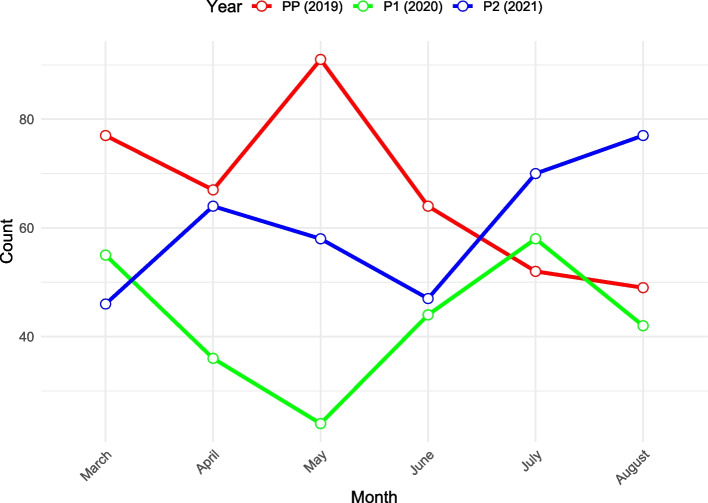
Monthly referral trends to the MDT during PP (2019), P1 (2020) and P2 (2021) phases

**Fig. 2:**
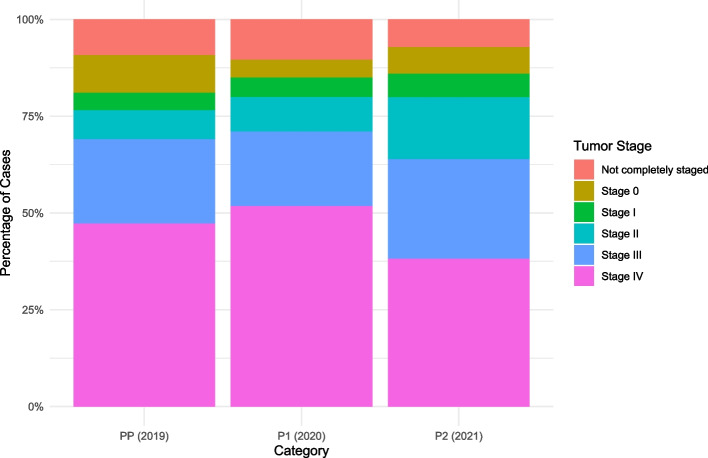
Comparison of tumour stages among patients in PP (2019), P1 (2020) and P2 (2021) phases

The mean age of patients during these periods was consistently around 71 years (2019: 71.03, 2020: 71.27, 2021: 71.23), with the differences being statistically non-significant (*p* = 0.88). The time from diagnosis to the first decision of treatment modality varied notably across these periods. During P1, this timeframe was significantly shorter (12.58 days) compared to both the PP (17.33 days) and P2 periods (27.33 days), with a *p*-value of > 0.001. Figure 1 provides insight and illustration into the monthly referral trends to the MDT during the PP (2019) P1 (2020), and P2 (2021) Phases. The distribution of tumor stages among patients in each period can be visualized in Fig. 2.

### Decision-making in MDT

Table 2 elucidates the variations in treatment decisions, encompassing the intent, reasons for chosen intent, and the initial treatment modalities. The analysis revealed no significant change in the overall treatment intent (i.e. curative vs. palliative) throughout the study period (*p* = 0.14). Furthermore, there were no significant variations in the reasons underlying these treatment intents.

**Table 2:** Analysis of treatment decision-making and management strategies in oesophagogastric cancer across PP (2019), P1 (2020) and P2 (2021) phases

	PP (2019)	P1 (2020)	P2 (2021)	Total
Intent of treatment (*p*-value = 0.1393)				
Curative	155 (38.8%)	81 (31.3%)	126 (34.8%)	362 (35.5%)
Palliative	245 (61.2%)	178 (68.7%)	236 (65.2%)	659 (64.5%)
Reasons for treatment intent (*p*-value = 0.1919)				
Potentially curative disease (Curative intent)	155 (38.8%)	81 (31.3%)	126 (34.8%)	362 (35.5%)
Advanced disease (Palliative intent)	264 (42.0%)	127 (49.0%)	279 (49.4%)	470 (46.0%)
Poor fitness (Palliative intent)	73 (18.2%)	47 (18.1%)	52 (14.4%)	172 (16.8%)
Patient Choice (Palliative intent)	8 (2%)	4 (1.5%)	5 (1.4%)	17 (1.7%)
Modalities were chosen as the first treatment (*p*-value = 0.006497)				
Best Supportive care	118 (29.5%)	71 (27.4%)	99 (27.3%)	288 (28.2%)
Palliative chemotherapy	114 (28.5%)	98 (37.8%)	125 (34.5%)	337 (33%)
Radiotherapy	13 (3.2%)	8 (3.1%)	11 (3%)	32 (3.1%)
Endoscopic treatment	42 (10.5%)	14 (5.4%)	24 (6.6%)	80 (7.8%)
Neoadjuvant chemotherapy	49 (12.2%)	33 (12.7%)	50 (13.8%)	132 (12.9%)
Neoadjuvant chemoradiotherapy	8 (2%)	2 (0.8%)	2 (0.6%)	12 (1.2%)
Radical chemoradiotherapy	45 (11.2%)	24 (9.3%)	27 (7.5%)	96 (9.4%)
Surgery alone	10 (2.5%)	6 (2.3%)	24 (6.6%)	40 (3.9%)
Watch and wait	1 (0.2%)	3 (1.2%)	0	4 (0.4%)
Rates of curative surgery (*p*-value = 0.1463)				
For Curative surgery	67 (16.8%)	41 (15.8%)	77 (21.3%)	185 (18.1%)
Not for surgery	333 (83.3%)	218 (84.2%)	285 (78.7%)	836 (81.9%)

A notable finding was the change in the first-line treatment modalities, demonstrating statistical significance (*p* = 0.006). Regarding surgical planning, the rate of patients with initial plans for curative surgery did not differ significantly across the years (*p* = 0.15). However, the time from diagnosis to surgery was significantly reduced P1, averaging 130.8 days, compared to 162.5 days during PP and 178.9 days in P2 (*p* > 0.001), indicating a quicker progression to surgical intervention during P1.

### Deviation from initial treatment pathway

A significant finding in this study was the change in treatment intent from curative to palliative, which showed a notable statistical difference across the groups (*p* = 0.014). In the P2 group (2021), 23 patients (6.4%) experienced a change in intent from curative to palliative. This rate was higher compared to the PP (2019) and P1(2020) groups; 9 patients (2.2%) and 9 patients (3.5%), respectively. The predominant reason for the change in treatment pathway in the P2 Group was disease progression, accounting for 22 patients (71%). This contrasts with the PP (2019) Group, where disease progression was the reason for changing the treatment pathway in 10 patients (41.7%) and the P1 (2020) group, where it accounted for 6 patients (54.5%) (*p*-value = 0.1464).

### Survival

**Fig. 3:**
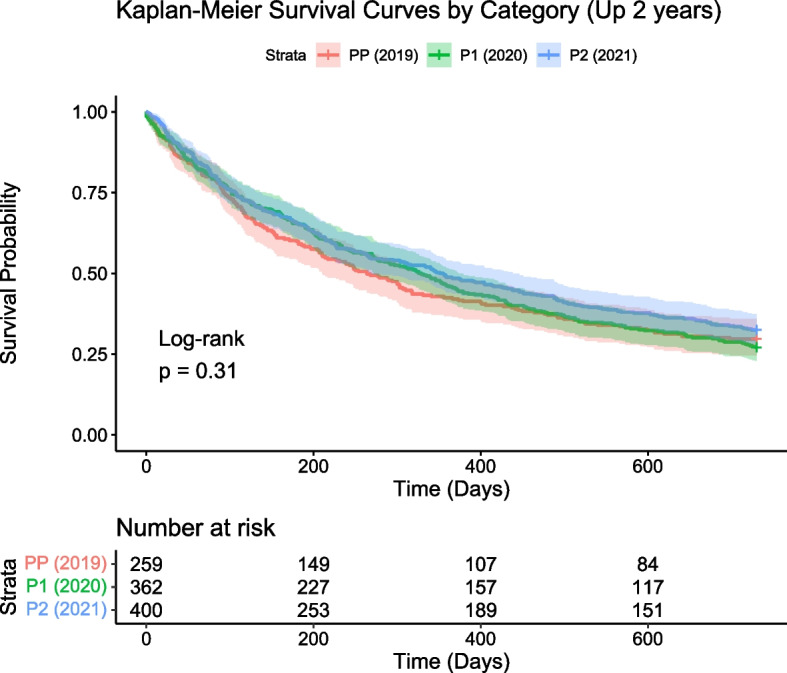
Kaplan–meier survival analysis for PP (2019), P1 (2020) and P2 (2021) patient groups

With regards to survival the PP group (2019) had a two-year survival rate of 32.5% (130 patients), while the Pandemic 1 g roup (2020) and Pandemi 2c group (2021) had survival rates of 29.7% (77 patients) and 27.1% (98 patients) respectively. These variations were not statistically significant (*p*-value = 0.2624), indicating that the pandemic's impact on the two-year survival of oesophagogastric cancer patients was not substantial within the scope of this study. Figure 3 presents a further analysis of survival probabilities, which illustrates a Kaplan–Meier curve with an accompanying number at-risk table. The Kaplan–Meier analysis yielded a *p*-value of 0.31, reaffirming that the differences in survival probabilities among the groups were not statistically significant.

### Postoperative pathology

**Fig. 4:**
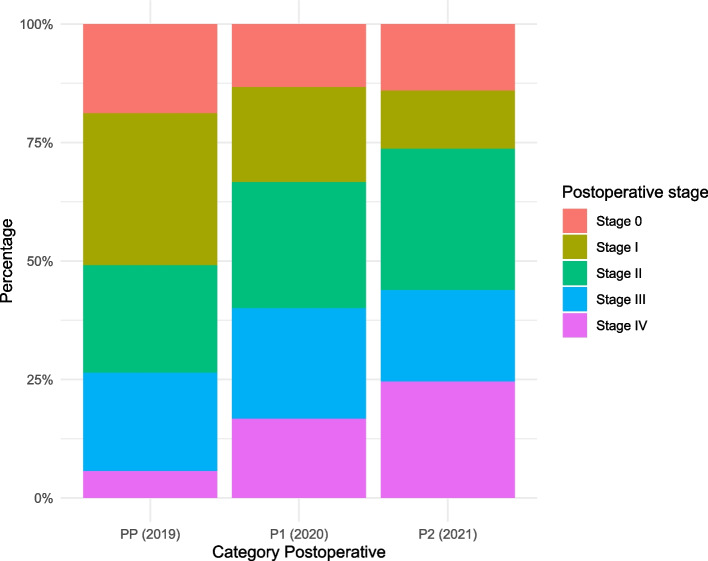
Pathological staging distribution, showing a notable increase in stage 4 tumors in the P2 (2021) group

There was a *tendency* towards more aggressive tumour characteristics in the P2 (2021) group, including an increased incidence of positive lymph node harvest and generally more advanced stages of cancer (Detailed in Table 2 in the appendix). Despite these observations, tumour staging differences were not statistically significant (*p*-value = 0.11). However, the increased number of specimens with positive resection margins in the P2 group (*n* = 20, 35.1%), compared to the 2019 PP (*n* = 8, 15.1%) and 2020 P1 (*n* = 6, 20%) groups, (*p*-value of 0.04), further indicates the more aggressive nature of tumours in this cohort. Figure 4 details the differential staging across all categories, highlighting a higher prevalence of stage 4 tumours in the 2021 Pandemic 2 group. A survival analysis of patients who underwent surgery indicated no statistically significant difference in survival propability across the groups (*p*-value = 0.59).

## Discussion

In this study, we aimed to investigate the impact of the COVID-19 pandemic on the diagnosis, decision-making, and management of patients with oesophagogastric cancer, covering three distinct periods – Pre-pandemic: PP (2019), Pandemic 1: P1 (2020), and Pandemic 2: P2 (2021). Our analysis provided a unique comparative perspective, revealing key findings such as a significant decrease in patient referrals during the peak pandemic, a consistent demographic profile across periods, quicker times from discussion to treatment in the initial phase of the pandemic, and notable changes in treatment modalities. Additionally, we observed a trend towards increased deviation to palliative intent after initially considering curative intent in the P2 group, primarily driven by disease progression. A further significant observation was the emergence of more aggressive tumour characteristics, particularly in the P2 (2021) group, albeit without a statistically significant difference in two-year survival rates among the groups. The higher R1 resection rate in the P2 group was not associated with changes in surgical techniques, as the proportion of open and minimally invasive surgeries remained consistent across all study periods. This aligns with findings from our earlier work comparing surgical practices to other European centres during the pandemic [8]. The increase in R1 resections is likely attributable to a higher prevalence of advanced-stage (T4) tumours in P2, reflecting delays in diagnosis and presentation rather than alterations in surgical practice. These findings underscore the complexities and adaptations in oesophagogastric cancer care during a global health crisis and highlight the pandemic's potential influence on tumour behaviour.

The significant decrease in MDT referrals during the peak of the COVID-19 pandemic, as observed in this study, aligns with global trends. Numerous studies conducted during the first year of the pandemic have consistently reported a decline in new patient referrals and diagnoses [9–14]. This trend, as evidenced by our findings, highlights the profound impact of the pandemic on healthcare access and patient engagement. The partial recovery in referral rates during the P2 period, while indicative of a return towards normalcy as pathways were reestablished, also suggests lingering effects of the pandemic on healthcare systems. These patterns underscore the need for further exploration into how healthcare disruptions, such as global pandemics, can significantly alter patient pathways and access to care. The reduction in referrals during the pandemic's peak can largely be attributed to reduced endoscopy services [15, 16] and constraints in primary care assessments for cancer, influenced by resource reallocation and patient reluctance to seek care amid COVID-19 concerns [11]. Additionally, the increased vulnerability of cancer patients during the pandemic may have led to higher mortality rates before diagnosis [17–19].

Contrary to delays in patient pathways suggested by the "National Oesophago-Gastric Cancer Audit: State of the Nation Report, 2020–2022 patient cohort," [20] a trend corroborated by other studies and reports [2, 21–24], our research indicates an accelerated decision-making process during the pandemic's peak. Similarly, the time from diagnosis to surgery decreased during the Pandemic 1 timeframe. Complementing our findings, an Irish study confirmed the feasibility and safety of continuing surgical resection for oesophageal cancer during the pandemic, reflecting effective management strategies in this challenging period [4]. Whilst numerous studies report the delays in patients being treated for cancer during the pandemic [9] we postulate that with fewer non-urgent presentations or elective surgeries being performed [2] the availability of resources for patient with a diagnosed cancer may have resulted in more availability for cancer care. Additionally, the implementation of safety measures to quickly restart major surgeries during the pandemic likely played a role in expediting treatment decisions [8, 25].

In our study, while there was no significant change in overall treatment intent (curative vs. palliative) across the pandemic periods, we observed a notable shift in first-line treatment modalities, particularly an increase in palliative chemotherapy and decrease in endoscopic modalities during the peak pandemic. This change likely reflects not only adaptive strategies to healthcare constraints but also compliance with new national guidelines developed in response to the pandemic [6, 15].

The increase in deviation from initially curative to palliative pathways in the P2 (2021) period underscores a significant trend towards more advanced disease presentations. This pattern is likely a consequence of the diagnostic delays experienced during the pandemic's peak. Such a rise in deviation highlights the prolonged effects of healthcare interruptions, emphasizing the necessity of continuous and efficient cancer diagnosis and management, particularly in the face of global health emergencies.

Our analysis showed no significant differences in survival rates among the three studied periods. This lack of statistical significance is seemingly at odds with expectations based on the pandemic's broader impact, as reported in this and other studies [26] may be ascribed to the efficient and continuous provision of oesophagogastric cancer services. Additionally, the age and vulnerability of patients, factors potentially leading to higher mortality before diagnosis independent of cancer progression, might elucidate this unexpected trend in survival outcomes.

To our knowledge, no prior studies directly link the delays in diagnosis caused by the COVID-19 pandemic to an observed increase in tumour stage, as indicated by the higher incidence of positive resection margins in the P2 (2021) group. While our findings did not demonstrate statistical differences in overall survival or staging, the apparent increase in tumour stage could potentially be attributed to delays in patients presenting to healthcare systems and, subsequently, delayed diagnoses. Considering the specific context of oesophagogastric cancer, further research is warranted to explore whether similar trends are evident in other cancer types or healthcare settings. This may uncover significant effects that were not captured in our study.

## Strengths and limitations

We utilized a comprehensive retrospective cohort design to assess the impact of the COVID-19 pandemic on oesophagogastric cancer care, analyzing patient data across three different periods. Conducted at a major UK tertiary referral EG center, our study is significant due to its large and demographically diverse sample size, enhancing the generalizability of our findings within similar healthcare settings. The study is unique in its scope and scale, the granularity of data it has captured, providing valuable insights for healthcare planning and response in future crises. While the findings from a single center may not fully represent other healthcare environments, they offer a detailed perspective on pandemic-related changes in cancer care. The two-year follow-up, although insightful, may not reveal longer-term outcomes; however, given the minimal differences in patient characteristics, especially consistent age across groups, and the nature of oesophagogastric cancer pathology, extended follow-up may not significantly alter the conclusions drawn.

## Conclusion

This study reveals how the COVID-19 pandemic significantly impacted oesophagogastric cancer care, with a notable reduction in patient referral rates during the initial pandemic and increase in more advanced disease later. Our findings highlight accelerated treatment decision-making during the initial phase of the pandemic and standard of care treatment was largely maintained with no significant difference in 2 year survival across the groups. These insights, within the constraints of a single-center study, provide valuable lessons for healthcare systems in managing cancer care during global health emergencies.

## Data Availability

No datasets were generated or analysed during the current study.

## Appendix 1

### Study collaborators

Surgery

- Khurshid Akhtar
- Naheed Farooq
- John Saunders
- Arfon Powell
- Javed Sultan
- Ram Chaparala
- Alan Li
- Chris Grocock

Medical Oncology

- Wasat Mansoor
- Richard Hubner
- Tom Waddell

Clinical Oncology

- Ganesh Radhakrishna
- Lubna Bhatt
- Hamid Sheikh

Gastroenterology

- Mark Kelly
- Robert Willert
- Hui Lee
- Neeraj Prasad
- Roger Prudham
- Yeng Ang
- James Britton

Pathology

- Michael Scott
- Anna Davenport
- Sue Pritchard
- Roger Hunt
- Nicholas Mapstone
- Lucy Foster
- Stephen McGrath
- Jamil Choudhury

Radiology

- Velauthan Rudralingam
- Haider Alwan-Walker
- Amir Mohammad
- Anas Hattab
- Ajay Tokala
- Abdulrahman Omer
- Neal Townsend

Anaesthesia & Critical Care

- Ravin Mistry
- Fiona Wallace

## Appendix 2

### Tables

**Table 3:** Appendix deviation from initial treatment pathway in Oesophagogastric cancer patients

	PP (2019)	P1 (2020)	P2 (2021)	Total
Did surgery happen? (*p*-value = 0.3864)				
Surgical intervention	54 (13.5%)	33 (12.7%)	59 (16.3%)	146 (14.3%)
No Surgical intervention	346 (86.5%)	226 (87.3%)	303 (83.7%)	875 (85.7%)
Operations performed (*p*-value = 0.1729)				
Oesophagectomy	37 (68.5%)	19 (57.6%)	36 (61%)	92 (63%)
Gastrectomy	16 (29.6%)	12 (36.4%)	22 (37.3%)	50 (34.2%)
Palliative	1 (1.9%)	2 (6.1%)	1 (1.7%)	4 (2.7%)
Change intention from curative to palliative (*p*-value = 0.01381)				
Yes	9 (2.2%)	9 (3.5%)	23 (6.4%)	41 (4%)
No	391 (97.8%)	250 (96.5%)	339 (93.6%)	980 (96%)
Change in modality with the same intent (*p*-value = 0.05055)				
Yes	15 (3.7%)	2 (0.8%%)	8 (2.2%)	25 (2.4%)
No	385 (96.3%)	257 (99.2%)	354 (97.8%)	991 (97.1%)
Reasons for deviation from pathway (intention and/or modality) (*p*-value = 0.1464)				
Disease progression	10 (41.7%)	6 (54.5%)	22 (71%)	38 (57.6%)
Incomplete resection	2 (8.3%)	2 (18.2%)	1 (3.2%)	5 (7.6%)
Patient choice	1 (4.2%)	1 (9.1%)	2 (6.5%)	4 (6.1%)
Patient deconditioned	11 (45.8%)	2 (18.2%)	6 (19.4%)	19 (28.8%)
Total	24 (36.4%)	11 (16.7%)	31 (47%)	66

**Table 4:** Appendix comparative histopathological findings post-resection across PP, P1 and P2 groups, highlighting tumour aggressiveness and staging

	PP (2019)	P1 (2020)	P2 (2021)	Total
Postoperative pathology T stage (*p*-value = 0.1359)				
T0	10 (18.9%)	5 (16.7%)	11 (19%)	26 (18.4%)
T1	1 (1.9%)	0	0	1 (0.7%)
T1a	2 (3.8%)	3 (10%)	2 (3.4%)	7 (5%)
T1b	14 (26.4)	3 (10%)	9 (15.5%)	26 (18.4%)
T2	6 (11.3%)	3 (10%)	4 (6.9%)	13 (9.2%)
T3	20 (37.7%)	11 (36.7%)	23 (39.7%)	54 (38.3%)
T4	0	0	1 (1.7%)	1 (0.7%)
T4a	0	5 (16.7%)	7 (12.1%)	12 (8.5%)
T4b	0	0	1 (1.7%)	1 (0.7%)
Numbers of lymph nodes harvested (*p*-value = 0.4974)				
Mean	28.49	30.87	29.44	
Numbers of Positive lymph nodes harvested (*p*-value = 0.1124)				
Mean	1.19	2.68	2.98	
Post-operative pathology Staging (*p*-value = 0.1064)				
Stage 0	10 (18.9%)	4 (13.3%)	8 (14%)	22 (15.7%)
Stage 1	17 (32.1%)	6 (20%)	7 (12.3%)	30 (21.4%)
Stage 2	12 (22.6%)	8 (26.7%)	17 (29.8%)	37 (26.4%)
Stage 3	11 (20.8%)	7 (23.3%)	11 (19.3%)	29 (20.7%)
Stage 4	3 (5.7%)	5 (16.7%)	14 (24.6%)	22 (15.7%)
Resection margins R0/R1 (*p*-value = 0.04176)				
R0	45 (84.9%)	24 (80%)	37 (64.9%)	106 (75.7%)
R1	8 (15.1%)	6 (20%)	20 (35.1%)	34 (24. %)
